# Disrupting Intergenerational Maternal Maltreatment in Middle Childhood: Therapeutic Objectives and Clinical Translation

**DOI:** 10.3389/fpsyt.2018.00623

**Published:** 2018-11-27

**Authors:** Jackie Amos, Leonie Segal

**Affiliations:** ^1^Center for Population Health Research, University of South Australia, Adelaide, SA, Australia; ^2^Centacare Catholic Family Services, Adelaide, SA, Australia

**Keywords:** maternal/child maltreatment, middle childhood, P-PACT, theory-aligned attachment, trauma, family therapy

## Abstract

**Background:** Child Maltreatment is a concerning worldwide problem. The population of distressed mothers with their highly disturbed children, in middle-childhood, often present to child and adolescent mental health services (CAMHS). Finding effective therapies for this population has proved elusive. This led the authors to undertake a theory-driven research program to better understand intergenerational child maltreatment from a clinical perspective, in order to determine how best to treat the entrenched distressing and destructive behaviors.

**Methods:** The model describing the mechanisms of the intergenerational transmission of maternal maltreatment is briefly described, from which the objectives of any effective treatment for these mothers and children are derived. A clinical model for achieving the therapeutic objectives is then elucidated.

**Findings:** Core objectives of therapy are; first to support the mother and child to develop differentiated senses of self and, second to disrupt a relationship style built on a competitive dominance and submission hierarchy, replacing it with a hedonic (cooperative and compassionate relationship style). This requires a deep healing of the mother and child's trauma histories. A clinical operationalization of these objectives, through a novel dyadic psychotherapeutic protocol, Parallel Parent and Child Therapy is described, which addresses the therapeutic objectives while attending to the safety of mother and child.

**Interpretation:** This research contributes to a better understanding of the components of effective treatment in what is a notoriously hard to treat population. It also illustrates the value of clinically informed theory development in understanding and refining treatment strategies for highly distressed and distressing populations.

## Background

Chronic exposure to childhood adversities, including maltreatment severely compromises development (1, 2) and has far reaching implications for the health and mental health of adult survivors (3), ultimately trapping some families in intergenerational cycles of disadvantage (4, 5). For child and adolescent mental health services (CAMHSs) and intensive family support services (IFSS) the situation is apparent on a daily basis. Mothers (parents) present their children to CAMHS for treatment of the child's disturbed behaviors. They also form the dominant case load for IFSS. A common feature is the highly disturbed mother child relationships, and the limited capacity of the mothers to reflect on the contribution of these disturbed relationships and their chaotic lives to the children's behavioral and emotional difficulties. Services are struggling to respond.

In an effort to determine how best to work with this population, a theoretical, deductive analytic-conceptual research program was commenced, using a formal theory-building approach (6, 7). The aim was to better understand why and how the distressing situation for these families develops, synthesizing existing theoretical, and empirical research into a clinically informed model.

The focus of our theoretical enquiry was the inter-generational transmission of relational trauma, neglect and abuse in infancy, and the effects in middle childhood, adolescence, and adulthood. This research yielded a published theoretical model of maternal maltreatment that affords a clear understanding of what is happening in these families and what drives apparently aberrant behaviors (8, 9).

After construction, the model was used to predict the fundamental objectives of any effective treatment for these families. The model and these objectives are briefly described below, to provide a context for the second part of the manuscript, where the authors analyse, in the light of the proposed theory, the unique use of the one-way screen in the dyadic psychotherapeutic protocol, Parallel Parent and Child Therapy (10, 11). The purpose of this analysis was primarily to propose a mechanism of action of the one-way screen, identified as an instrumental element of the therapy.

## Model of maternal maltreatment

The model of intergenerational maternal maltreatment that we developed (8, 9) is integrative in intent and synthesizes research from a number of academic discourses, with a strong focus on evolutionary theory and ethology. A bio-behavioral explanation of personality development is central to the model, which combines attachment theory, the role of two sets of mammalian defenses against threat—individualistic anti-predator defenses and group living defenses (12, 13) and the theory of structural dissociation of the personality (14). Other influences include cognitive behavioral theories of symptom formation in trauma syndromes (15), interpersonal neurobiology (16, 17), and exposure as a core component of trauma specific treatment protocols (15).

The theoretical model identifies trauma as the most useful lens for thinking about how to intervene with these families (8, 9, 18).

In the model, the primary traumatic injury, relational trauma (17, 19), is the result of a mother being both helpful and endangering, from the infant's perspective; failing to protect her infant from becoming overwhelmed by fear and distress and failing to meet and mirror her infant's unique emotional communications. The published model incorporates empirical research that examined the relationship between five broad categories of disrupted emotional communication displayed by mothers and their toddlers, in the Strange Situation, at 18 months of age and attachment disorganization (20). Results indicated that high levels of disrupted emotional communication predicted attachment disorganization, with emotional withdrawal by mothers being the most predictive (21). Beebe et al.'s (22) microanalytic studies of interactions between mothers and infants at 4 months of age, who later develop disorganized attachment-caregiving relationships confirms the centrality of relational trauma to all forms of maltreatment. This work also confirms that maternal emotional withdrawal, facial immobility, and incongruent responses to infants' emotional communications are particularly deleterious.

Extending current understandings, it is proposed in the model that an infant in this situation not only experiences fright without solution, secondary to the failure of their mother to meet basic care needs, combined with the atypical forms of emotional communication (23) but also shame without solution secondary to the resulting failure of mirroring (18). This toxic combination of unresolvable distress, referred to in the model as terrified shame without solution, is a seriously disruptive and unmanageable somatic-emotional experience. As with all experiences encountered in early infancy, terrified shame without solution is encoded unconsciously in implicit memory (24, 25) and remains unprocessed because the required relational support is not available (18). Accepting that the roots of self-experience are, at a physiological level, underpinned by the smooth functioning of the autonomic nervous system (ANS) (26) and that shame is particularly disruptive to the operation of the ANS, terrified shame without solution is proposed as the basic biological substrate for subjective experiences of emptiness and annihilation anxiety (27). Beebe and colleagues' interpretation of the impact on infants, of the patterns of communication between mother and infant, described in their research is that the infant develops models of not being “sensed, known or recognized by their mothers” (22), and they therefore struggle to know themselves. This is a logical outcome of the shame related experiences of emptiness and annihilation. In addition, infants in these disturbed relationships fail to experience contingencies between their emotional expressions and the mother's response, compromising their developing sense of agency in relationships (28).

Initially, the infant's distress activates both the infant's attachment system and their individualistic mammalian defenses: avoidance, attentive immobility (hyper vigilance), flight, fight, and tonic immobility (or playing dead) (12, 18). Activation of the attachment system motivates the infant to seek proximity to their attachment figure, while the defensive systems motivate escape. This gives rise to the approach avoidance dilemma, that characterizes a motivational systems conceptualization of disorganized attachment-caregiving relationships (8, 14, 29). Infants switch between defensive systems, “looking” for a solution. These strategies are ultimately ineffective, as the infant is unable to resolve the approach avoidance dilemma.

The terrified shame without solution (and the ineffective responses related to the anti-predator defensive solutions) are dissociated into an organizational structure of mind, referred to in this model as the Attachment Related Dissociative Part of the Personality (ARDP) (8), which is separated from the rest of the developing personality (8, 14). For the mother, who, through her own childhood maltreatment history has been unable to integrate ARDP (which is almost certain in the absence of effective interventions), ARDP will be activated by the presence of her emotionally and physically dependent infant, flooding the mother with unmanageable somatic/emotional distress. The mother consciously attributes the distress to the presence of her infant (source attribution error) (30), not recognizing the unconscious links to the relational trauma, neglect and abuse experienced in her early relationships with her primary caregiver(s). Since the distress (unconditioned stimulus) is paired with the particular communications and behaviors of her infant, these communications become classically conditioned signals that the aversive inner experience of ARDP activation is about to overwhelm her (14). In the model, the hypothesis is advanced that the mother attempts to re-stabilize her emotional distress, by “acting on” the infant (the perceived source of distress) in automatic, unconscious and defensive ways, explaining acute episodic maltreatment (8). Over time, the mother and child become potent signals for the imminent reactivation of ARDP in each other, a volatile and potentially dangerous situation.

The work of Michael Chance (31, 32) and colleagues, and Kortmulder and Robbers (33) which details different forms of group relationships that support threat management within groups of mammals, provides a new way of understanding how the intense emotional distress of mother and child in the presence of each other is eventually stabilized. The solution involves spontaneous and repeated switches to a competitive form of relational organization, agonic mode, where strict adherence to aggressive dominance and submission hierarchies, decreases ongoing threat by increasing predictability (9). In agonic mode, emotional closeness is sacrificed in the service of maintaining proximity while reducing risk of lethal engagement (33). Attention is focused externally on the other, compromising the child and the mother's capacity to develop a sense of self, based on distinctive inner experience. Instead the aggressive, dominant and the submissive, appeasing positions become the basis of self-systems that accommodate to the externally derived needs of the agonic system (9, 18). The mother and growing child become enmeshed, functioning as two parts of a single defensive self-system where each experiences the other as an object to be managed, rather than a unique subject requiring understanding (18). The individual in the dominant position disavows experiences of vulnerability, including fear and shame, which are then taken up and embodied by the individual in the submissive position (18). However, when they look to one another for confirmation of their identity, the dominant encounters an individual who is submissive and appeasing and vice versa, thus encountering another, who is not recognizably similar (18). The subjective experience is one of terrible isolation from the rest of the human family (18). The switch to agonic mode is an automatic action of the procedural or unconscious mind (18, 25).

This conceptualization offers a cogent explanation for the unpleasant controlling caregiving and controlling hostile punitive adaptations observed in middle childhood (34), and the chronic hostility and helplessness described in the Hostile/Helpless coding system on the Adult Attachment Interview (35). An interesting link can be postulated between agonic mode as conceptualized in this model and the failure to develop the capacity for mentalization, a well-accepted outcome of disturbed attachment-caregiving relationships (36). Mentalization refers to the capacity to think about the internal worlds of both self and others (37), something that is severely diminished in agonic mode where the other is often a problem to be managed in automatic ways, without reflection.

In essence, this is a sophisticated trauma model of symptom formation, resulting in a dyadic form of chronic interpersonal trauma (18). Enmeshment, competition, objectification, isolation, and maltreatment are all safety behaviors supporting mother and child to avoid ARDP reactivation. The mother's avoidance of ARDP prevents her from parenting in ways that optimize the child's development and the child's avoidance of ARDP leads to ongoing emotional and behavioral disturbances.

## Objectives of treatment

Three fundamental objectives of effective therapeutic intervention follow logically from the theoretical model. The first is to disentangle the mother and child from their shared, trauma-related, enmeshed defensive self-system by facilitating the processing of the terrified shame without solution (and ARDP). The second is to disrupt the mother and child's reliance on agonic mode, which underpins the competition, isolation, enmeshment, and objectification, and their failure to develop differentiated senses of self with agency. The third is to increase the mother and child's capacity to operate in an alternative cooperative relational mode, known as hedonic mode. Hedonic mode is typified by egalitarian cooperation between individuals, who, though not equal in status, power or resources, are equal in their humanity (9, 18, 33). In hedonic mode individuals are compassionate and empathic, recognizing one another as similar, but not the same, conditions that promote the development of a robust sense of self with agency (38).

### Core components of an effective therapeutic intervention

Evidence-supported treatments for posttraumatic states, including those resulting from chronic interpersonal traumatization, in adults and children, incorporate exposure as a core element (15). Hayes et al. (39) and Carey (40) assert that not only is exposure central to the treatment of posttraumatic states, but that exposure is a significant element of all effective psychotherapies:

“There seems to be an implicit consensus in the literature that facing, confronting or experiencing, becoming aware of, integrating or otherwise being exposed to, those experiences that one would otherwise avoid or not engage with is an essential component of successful therapies” [(40), p. 240].

In practice, exposure to avoided mental states is facilitated when avoidance mechanisms are disabled (response prevention). Intensity needs to be graded so that affect remains within the window of tolerance, and traumatic material needs to be processed (18, 41). Processing traumatic material involves, increasing affect recognition and affect tolerance (42, 43); organizing memories into coherent narratives, linking emotional memories to missing contextual information (43, 44) and challenging trauma related meanings and beliefs (45).

The effectiveness of exposure can be increased by inducing dissonance and constraining how it is resolved (46), and holding clients in emotional contact with internal conflicts, without providing solutions (40).

### Challenges to the effective implementation of exposure

Trauma is central to our model of intergenerational childhood maltreatment, but successfully applying the principles of exposure to the treatment of these families is technically challenging. Effective exposure to terrified shame without solution/ARDP is hindered by the strength, automaticity and unconscious nature of the avoidance. Furthermore, in agonic mode, conflicting states of mind are located in different individuals who function as two parts of a single defensive self-system, complicating the identification of internal conflicts (18).

Safety is extremely important. At home, acute maltreatment of the child by the mother restabilizes ARDP activation, at the expense of the child's safety. This must not occur in treatment (9). Further, the mother is a potent activator of ARDP in the child, and thus the child will require effective support to limit the emotional and behavioral dysregulation driven by ARDP activation (18). As the cycles of trauma reactivation are bi-directional, treatment must include mother and child, to prevent differences in individual progress undermining necessary changes in the relationship dynamic (18).

Processing of trauma related material is complex. ARDP is implicitly encoded prior to the development of language (8). Initial activation can be intense and stray outside of the window of tolerance. The context for the mother's early trauma is in her family of origin, but for the child the context in which ARDP was formed is also the current relationship. Early in treatment, the relationship is still unsafe for the child, which makes re-contextualization of the child's emotional memories from infancy more challenging (18).

### Meeting the objectives of therapy and achieving acceptable safety

Dyadic psychotherapy protocols designed to help distressed mothers and their infants and young children have been steadily increasing since the 1970s when Fraiberg et al. (47) suggested that a baby can be the object of maternal transference, and that focusing treatment efforts on this transference relationship would improve outcomes for vulnerable infants. Although not explicitly based in attachment theory, later researchers have highlighted the similarities between Fraiberg's approach to treatment and those based in attachment theory (48). Watch, Wait, Wonder (49), Circle of Security (50), Attachment Based Family Therapy (51), and Attachment and Biobehavioural Catch Up (52) are some of the protocols that have emerged since the early 2000's that are accumulating good evidence for their effectiveness. With the exception of Attachment Based Family therapy, which is designed to work with adolescents, these approaches commonly target parents, infants and children under the age of 5 years and require the mother and her infant/child to be able to be seen together in the same room.

Parallel Parent and Child Therapy (P-PACT) was developed in response to the clinical need for an intervention to help mothers and their older children, typically between 6 and 12 years of age—although the protocol can be used successfully with children as soon as they are verbal (Chambers and Foley, personal communication). A unique aspect of the P-PACT protocol is the way a one-way screen is used, to allow the mother to watch her child interacting with their therapist in the third stage of treatment. The one-way screen allows the protocol to be used where the levels of hostility, intrusiveness, or failure of responsiveness preclude joint work. These mothers typically have limited capacity to reflect on their own or their child's inner world. In early clinical experiences with P-PACT mothers often revealed, in Stage 3, ongoing neglect and abuse of their child, that their therapists had not been aware of until that time (8). Stage 3 is also the stage that mothers spontaneously identify as being pivotal in changing their understanding of their child and consequently their parenting responses (18).

P-PACT (10, 18) is now a semi-manualized dyadic psychotherapy, for which there is case study evidence that it can disrupt intergenerational cycles of maltreatment where the maltreatment is ongoing at the time of intervention (53). P-PACT requires two therapists, a playroom and an observation room connected by a one-way screen.

The purpose of Stages 1 and 2 is to develop an in-depth, and shared understanding of the intergenerational relational context of the mother—child relationship, which is essential to support Stage 3.

In Stage 1, Parallel Parent and Child Narrative (PPCN) (18), therapists focus on the story of the mother child relationship from conception to the present day. Ideally the mother and child are together in Stage 1 although this stage can be modified and conducted with the mother alone. The usual history taking process is slowed down considerably to allow for a detailed exploration of emotionally salient aspects of the mother's relationship with her fetus/infant/growing child and vice versa. To give a sense of the pace, it might take two, hour-long sessions to hear the story of the pregnancy and delivery. The therapists aim to explore the inner worlds of both the mother and her fetus/infant/growing child in relation to events in their relationship and to one another, bringing the inner experience of each into view for the other. In this stage therapists also use a relational interpretation, devised by Chambers (10), where existing understandings are reframed as understandable mistakes of meaning and blame, the good intentions behind hurtful actions are rigorously sought, and the situation is re-framed as sad.

An example of this form of narrative is taken from a PPCN story with a mother and her 7-year-old son. The mother experienced severe depression in the postnatal period. The mother identified that although she took care of her infant's physical needs, for much of the time when she was holding her infant she was staring blankly, with tears running silently down her face. The mother was deeply ashamed of her failure to respond to her infant son, who cried incessantly for the first months of his life, talking about herself as a bad and neglectful mother. Speaking for the baby, therapists identified that the infant may have made a mistake that his mother didn't love him, that he wasn't worthy of care and connection and that there must be something very wrong with him. Therapists highlighted the mother's good intentions in providing for her infant's physical needs, but also in attempting to hold and connect with him. The infant's good intentions included not giving up crying as a way of trying to reach his mother. Therapists talked about how nobody chooses to be depressed and how the mother sought help to get well, before pointing out that the depression had robbed the mother of the chance to be the mother that she wanted to be; and that the infant missed out on the care that he needed and deserved. Nobody's fault but desperately sad.

In Stage 2 the mother and the child work separately. The mother and her therapist explore in detail the mother's subjective experiences and internal working model(s) of attachment using the Adult Exploration of Attachment Interview (AEAI) (54). The mother and her therapist construct a genogram, with the mother in the position of child, and identify all of her caregivers. The second task is to construct what is referred to in this protocol as an inheritance map (internal working model) of the mother's relationship with each of these caregivers. This is achieved by reflecting with the mother on two questions: what did you learn from this caregiver about being a mother, and what did you learn from this caregiver about being a child. The mother is encouraged to tell any stories that come to mind, remembered or shared by family members. The key skill for the therapist is to turn the stories offered by the mother, into statements about caring and being cared for. The statements are recorded on a piece of paper with a line down the middle and one of the questions written at the top of each column, creating a set of maps that can be referred to in Stage 3 of the therapy. To illustrate, a mother told a story about how, as a child, she would be forced to visit her auntie's house with her parents every Sunday. She would be forced to dress in her best clothes, but then banished to the garden to amuse herself with her cousins, who wore old clothes and engaged in high energy outdoor games. She would then be chastised for getting her best clothes dirty. Some of the statements that the therapists recorded included, mothers control what children wear, mothers make children stand out from their cousins, mothers make it hard for children to play and have fun, and mothers don't understand children. From the child's perspective, statements included, children's wishes and needs are not important, children have to do as their mother tells them even when it leaves them lonely, and children are torn between pleasing their mother and having friends.

The primary purpose of Stage 2 for the child and their therapist is to familiarize the child with the playroom, explain what happens in Stage 3, introduce them to the one way screen and address any fear that they might have about being watched by their mother. The child and their therapist also begin to engage in non-directive play therapy. The therapist, keeping the PPCN story in mind, starts to construct an understanding of the child's subjective experiences of their relationship with their mother and their internal working models of attachment.

In Stage 3 the child and their therapist engage in child-led, non-directive play therapy, while the mother, supported by her therapist, watches silently through a one-way screen. The child is aware of their mother watching but cannot see her. The child and child's therapist control the duration of the looking, using a curtain that obscures the one-way screen and signals to the mother's therapist to turn off the sound connection. The period of looking rarely exceeds 20 min. Once the curtain is closed, the mother reflects, with her therapist, on the experience of watching her child. Similarly, the child's therapist helps the child to understand their experience of playing, exposed to the mother's gaze, and the impact of closing the curtain. At the end of the session all four participants are reunited in the observation room to share what they have learned and for the child's therapist to hand the child back into the mother's care.

Stage 4 closely resembles Stage 3, except the mother is gradually introduced into the playroom, creating opportunities for increasing intimacy.

### P-PACT: a sophisticated exposure and response prevention protocol for dyadic relational trauma

P-PACT is a complex treatment that works on multiple levels and, as explored below, when considered against the theoretically predicted objectives of effective therapies, looks to meet all of the objectives of treatment (18). This section of the manuscript, through the authors' synthesis of the theoretical model and clinical observations, proposes how P-PACT in general, and the one-way screen in particular, overcome the technical challenges highlighted above to simultaneously expose the mother and child to ARDP and support self-other differentiation.

### PPCN and the AEAI beginning the process of trauma exposure and self-other differentiation

PPCN and the AEAI are not the central focus of this manuscript, however they both include elements of exposure. PPCN is a narrative process that exposes both mother and child to emotional memories of difficult times. In the AEAI, the mother, again through storytelling, revisits her own distressing childhood. As the statements derived from her stories are recorded on inheritance maps, she is confronted with all of her internalized beliefs at one time, rather than sequentially as would ordinarily be the case. This increases the intensity of the exposure.

In addition, both of these stages demonstrate for mother and child what it means to think about each other in terms of mental states. In PPCN therapists speak for the baby in the child and the mother, pointing them toward the impact that their early emotional experiences have had on how they currently experience themselves and their relationship. In the AEAI, the process of the mother and her therapist working collaboratively to construct statements from her stories is also an exercise in developing the capacity for self-reflection. As this includes her stories about herself as a child, this stage begins to support the mother to consider her child offspring's inner reality. Although the form that these stages take differs from that advocated in Mentalization-Based Treatment (55), it is hypothesized that these stages in part work to increase the capacity for mentalization, in particular in the mother. In the language of this model, the focus on the mother and the child's distinctive inner world, is important in promoting self-other differentiation.

### The one-way screen, exposing the mother to ARDP

The one-way screen in Stage 3 supports the safe activation of ARDP in the mother in two ways. First, by physically separating mother and child, the enmeshment that characterizes agonic mode, is disrupted. Second, the mother must face the most potent signal trigger for ARDP, her child, whilst the one-way screen prevents her from acting on her child in unconscious defensive ways (acute maltreatment) or reinstating the aggressive dominance, submission hierarchies typical of agonic mode, a form of response prevention. The one-way screen in preventing any maternal responses to ARDP activation from impacting directly on the child in the therapy setting also provides the required safety for the child. The duration of looking allows the activation to build in intensity, until visible markers of ARDP activation can be observed.

Mothers initially show outward signs of avoidance, consistent with ARDP being successfully kept out of awareness. These signs include, looking but appearing to not notice the child or be affected by them, distracting themselves by trying to converse with their therapist or reaching for their mobile phone. At this stage, after the curtains close the mothers are often unable to report any physical or emotional responses to watching their child. Conversation focuses on the child's behavior across the week and the mother's dissatisfaction with the same.

With recurring periods of watching, the therapists begin to notice new and subtle changes in the mother's response. As reported by Amos [(18), p. 172], “the reactions range from slight changes in posture or facial expression, pallor, reddening of the face, alterations in depth and rate of breathing, to overt physical agitation, sighing, groaning, rocking, moving the chair, or turning anxiously or angrily to engage their therapist in conversation.” These reactions are thought to represent the beginnings of ARDP activation in the mother.

When the mother begins to become consciously aware of her distress, words arrive with an emotional force. Mothers typically say things like “I wanted to smash through the window and hit him,” “I just wanted to yell at her to stop,” “I wanted the ground to swallow me up,” “I wanted to run away” [(18), p. 173]; statements that reflect the desire for dominance or a collapse into submission, consistent with agonic mode.

The mother's therapist keenly observes the mother and child, taking particular note of what the child is doing when the mother begins to become activated. After the curtains are closed, the mother's therapist explores with the mother, her experience of watching her child. Using the observations made during the looking period and the information obtained in Stage 1 (PPCN) and Stage 2 (AEAI) of the process, the therapist can make an interpretation of the infantile origins of her distress, which corrects her source attribution error by connecting the distress with its original context. In this way beliefs about how mothers and children are supposed to be, that have their roots in ARDP can be reconsidered in the light of the experiences occurring during the “here and now” of the therapy.

One mother, who had been neglected and physically abused by her mother as an infant and young child, sexually abused at the age of 9 years and then accused of sexually corrupting a slightly older cousin, became agitated while watching her son attack a punching bag with a wooden stick. She moved between a freeze response where she sat with her hand half way to her mouth for up to thirty seconds, and agitated sighing. She then turned anxiously to her therapist before laughing in a hollow manner. In Stage 1, she had talked about having attempted to suffocate her infant son when he would not stop crying. In Stage 2, she revealed that she believed that she was dangerous to males. In her individual session after the closing of the curtains, the therapist wondered what having a male infant might have been like for her, given her belief about herself as dangerous to males and her experience of having been sexually abused. She broke down and sobbed that she had hated her baby for being a boy, something she stated that she had been unaware of until that moment. A verbatim transcript of the sessions that are summarized here is available [(18), appendices A, B, and C, p. 217–234].

When therapists are successful in maintaining the mother's focus on her unique, inner responses to her child, this promotes self-other differentiation. Emergent self-other differentiation often leads to marked changes in the mother's perception of her child. For example, mothers have said, “oh, he's just little,” “he's separate from me, he is his own person,” “she isn't evil she's just a little girl who likes to do puzzles like I did” [(18), p. 177]. This differentiation is initially extremely painful for the mother. One mother cried out that if she was separate from her son then she was “no one, less than a dog” [(18), p. 177]. Others report not knowing who they are, or feeling overwhelmingly lost and alone in the world.

When mothers integrate these powerful experiences, they can begin to develop empathy for their child's predicament, finding new effective ways to be with their child predicated on a genuine and felt understanding of their child's needs (18). This gives mothers a powerful experience of agency.

### The one-way screen, exposing the child to ARDP

An understanding of the importance of gaze in mother-infant relationships reveals how the one-way screen facilitates exposure to ARDP for the child.

Typically, new mothers gaze at their infants, to come to know their infant and infants use their mother's gaze to regulate arousal and to develop a sense of self, as their mother mirrors her infant's unique emotional experiences (56). Where this relationship is traumatizing, the quality and meaning of the mother's gaze is profoundly altered. During ARDP activation and acute maltreatment the mother's eyes communicate her own distressing emotions, no longer mirroring those of her infant. The absence of facial expression, in the blank dissociative stare or unmoving face of depressive misery, or the mother's chronically anxious demeanor may accompany emotional withdrawal and neglect. Where the mother's gaze is discordant with the infant's inner experience, it becomes linked, via classical conditioning, to powerfully discomforting experiences, becoming a potent trigger for ARDP in the child.

As the one-way screen prevents the child from seeing the mother, they cannot use her signals to guide their behavior. Without this they cannot maintain the externally defined equilibrium of agonic mode.

Children typically respond in number of different ways to playing in front of the one-way screen. They can be overtly anxious and distressed, dazed, and disorientated and unable to decide what to do, or become overactive. They may try to stop the mother watching by turning their back to the one-way screen, or build barriers and hide behind them. We hypothesize that these children have not yet switched chronically to agonic mode. Others are more obviously dominant. They “order the therapist to get games for them to play, play with their eye on wining, bending the rules to suit themselves. They strive to show off their prowess, treat the therapist with contempt, often humiliating them in front of the one-way screen with unflattering personal comments” [(18), p. 180]. The final group of children tries to please the therapist, trying to work out what the therapist likes to do, or by monotonous play scenes in which the child cares for the therapist. The latter two groups are exhibiting behaviors consistent with dominance, submission, and agonic mode.

Once ARDP is clearly activated, the child's therapist helps the child organize their somatic-emotional experience into a verbal narrative, making educated guesses about the child's inner experience, based on the PPCN story, observations from the child's play and from their bodily cues. The child's therapist also makes connections between past and present, enlarging the context for the child's emotional responses to their mother. Existing beliefs are also challenged, for example, the child's therapist uses the mother's engagement with treatment to challenge a typical trauma related belief of the child, that their mother does not care about them.

One 9-year-old boy spent many weeks attempting to dominate the therapist. He enjoyed bringing games from home so that he could demonstrate how much better he was than the therapist at the games. He imposed strict rules on the therapist, while he cheated at everything, and made contemptuous comments about how useless the therapist was and what a waste of time therapy had turned out to be. This changed quite abruptly one day when the time came to close the curtains. He began to wrap himself up in the curtains, he ran up and down in front of the window and when the therapist closed the curtains as per the protocol he fell to the ground. He slowly sat up and began to rip sheets of white paper into tiny pieces. He collected them in a plastic bucket and when it was full he stood on a chair and gently tipped the pieces out, all over the floor. The therapist reminded him of how they had learned in the PPCN story that his mother had often been unable to attend to him as a young infant, because of ill health, leaving him for many hours, alone, hungry and in need of a change of nappy. The therapist hypothesized that he might be trying to show her how he shattered into thousands of pieces at these times, and that he was still frightened that his mother would leave him to once again shatter, without hope of recovery. The child kept the pieces of paper and at a later date, when his relationship with his mother was much stronger and they had moved to stage 4, he spent a session taping them back together. He then used the newly created object as a mat on which he and his mother played games together.

The one-way screen also supports self-other differentiation for the child, by exposing them, simultaneously, to three “mirrors.” The first is the perceived mirror of the mother's hidden gaze, reinterpreted by the therapist as the mother's response to her own inner world rather than to her child. The second is the perfectly contingent reflection provided by the mirrored surface of the one-way screen, and the third is the sensitive and attuned mirroring provided by the child's therapist. All three sources of information are inconsistent with the confusing and frightening early experiences of failed mirroring. This induces dissonance, which, if successfully resolved, provides the child with a developmental second chance.

In the play therapy the child is given the lead, providing them with an opportunity to develop a sense of agency.

### Using cooperation (hedonic mode) to enhance exposure

In addition to enmeshment; competition, objectification, and isolation are other features of agonic mode. Countering these features is instrumental in supporting effective exposure. Providing a rigorously cooperative relational context, serves well-understood functions such as building trust and minimizing shame, but also counters objectification, isolation and competition. Destabilizing agonic mode, whilst simultaneously modeling the features of hedonic model, supports the shift from an agonic to hedonic relational mode.

A rigorously cooperative relational treatment context is achieved, through the use of a particular therapeutic stance and a particular method of delivering interpretations (10, 18). P-PACT therapists are (and must be) generous, compassionate, and non-judgmental. They immerse themselves in the mother's and the child's experiential worlds, offering predictable, sensitive, attuned and deeply empathic responses. The mother and child have the opportunity to relate to therapists, who try their best to understand them, rather than dominate, or submit to them. When therapists are able to understand and then convey their understanding verbally and non-verbally, the mother and child begin to know at an explicit and implicit level, that they are not beyond the reach of human recognition. This provides a powerful counterpoint to previous experiences of isolation.

In addition, underlying the two poles of agonic mode, both mother and child are struggling with terrified shame without solution. Consequently, there will be discernible similarities between them, an assertion that is borne out by clinical experience. When the mother and child's mutual fears of vulnerability and connection are highlighted; and how these fears fuel many seemingly disparate responses to one another are explored, their sense of isolation is significantly reduced.

The therapists' focus on understanding the mother and child's personal and particular experiential worlds is the natural antidote to the objectification that typifies agonic mode.

### Therapist transparency: countering competition

Competition, in agonic mode, rests, in part, on the need for the individuals to compete for scarce resources, such as positive attention, validation, and confirmation (57). Where individuals rely on dominance and submission to navigate their relationships, the dominant individual has greater or exclusive access and entitlement to these resources.

Maintaining a cooperative relational environment requires therapists to avoid moving into dominance or submission. This injunction includes therapists' relationships with mother and child but also their relationships with one another. Therapists must distinguish between being an expert (a dominant position) and having expertise (a cooperative position) to avoid competition between them and the mother and child about who is right.

Therapists need to be transparent when offering possible interpretations, communicating a sense of open inquiry, supporting two aims. First, each therapist can easily understand how his or her co-therapist is thinking about what is happening during treatment. Second, mothers and children have the opportunity to consider the therapists reasoning and decide for themselves whether or not to accept interpretations. This helps reduce the risk of clients appeasing therapists by taking on their viewpoint, or rejecting it out of hand, both of which would be typical of agonic mode.

Practically this involves therapists separating out what they are observing in therapy from the inferences that they are making about what is observed, so that they can clearly describe the pathway by which they have constructed their inferences. For example, making the difference between an observation of aggressive behavior and the interpretation of this as an expression of unmet need overt, and explaining the rationale (the PPCN story tells us that aggression has been a good way for the child to get the attention of an overwhelmed mother). This approach not only allows everyone to weigh up the validity of the idea, but again helps the mother and child to see how the therapist uses a reflective process to arrive at their conclusions. This further increases the mother and child's capacity to take a mentalizing stance toward one another.

## Conclusion

The theoretical model presented in this manuscript extends our understanding of how to effectively and safely incorporate exposure, a central component of most evidence-supported treatments of trauma into the treatment of implicitly encoded, dyadic interpersonal traumatization. P-PACT, a novel dyadic therapy, meets the theoretically predicted objectives of treatment. Stages 1 and 2 provide a rich understanding of the intergenerational relational context in which the mother child relationship is embedded. The one-way screen ensures adequate activation of unconscious traumatic material, maximizes safety for the child and supports self-other differentiation. A central requirement is to embed exposure in a cooperative (hedonic) relational context. This not only creates safety and reduces the risk of re-traumatization but paradoxically increases the exposure component of the treatment.

This research program demonstrates the utility of rigorous theoretical research, especially when informed by rich clinical experience. The process of gaining a deeper understanding of the underlying mechanisms, which create and perpetuate distress in severely compromised mother-child relationships, provided greater clarity about the objectives of therapy, and supported a new understanding of a promising clinical solution. More generally, theory-based research, simultaneously drawing on clinical experience and a sound understanding of the literature, offers a pathway for identifying effective solutions for the highly troubled families who come to CAMHS and IFSSs for help, and the therapists who wish to help them.

## Author contributions

JA had the primary role in exploring the impact of the theoretical model of maternal maltreatment on the objectives of therapy and testing this against P-PACT, her clinical therapeutic model and prepared the first draft of the manuscript. LS assisted in describing the meaning of the theoretical model for the objectives of therapy, testing P-PACT against those objectives and contributed to manuscript development and revision.

### Conflict of interest statement

The authors declare that the research was conducted in the absence of any commercial or financial relationships that could be construed as a potential conflict of interest.
